# Epi-miRNAs: Regulators of the Histone Modification Machinery in Human Cancer

**DOI:** 10.1155/2022/4889807

**Published:** 2022-01-11

**Authors:** Deniz Mortazavi, Behnoush Sohrabi, Meysam Mosallaei, Ziba Nariman-Saleh-Fam, Milad Bastami, Yaser Mansoori, Abdolreza Daraei, Sepideh Zununi Vahed, Shadan Navid, Zahra Saadatian, Tannaz Jamialahmadi, Yong Teng, Amirhossein Sahebkar

**Affiliations:** ^1^Koç University Research Center for Translational Medicine, Istanbul, Turkey; ^2^Department of Biology, Faculty of Sciences, Arak University, Arak, Iran; ^3^Department of Genetics and Molecular Biology, School of Medicine, Isfahan University of Medical Sciences, Isfahan, Iran; ^4^Women's Reproductive Health Research Center, Tabriz University of Medical Sciences, Tabriz, Iran; ^5^Non Communicable Diseases Research Center, Fasa University of Medical Sciences, Fasa, Iran; ^6^Department of Medical Genetics, School of Medicine, Babol University of Medical Sciences, Babol, Iran; ^7^Kidney Research Center, Tabriz University of Medical Sciences, Tabriz, Iran; ^8^Department of Anatomy, Faculty of Medicine, Social Determinants of Health Research Center, Gonabad University of Medical Science, Gonabad, Iran; ^9^Department of Physiology, Faculty of Medicine, Infectious Diseases Research Center, Gonabad University of Medical Sciences, Gonabad, Iran; ^10^Department of Nutrition, Faculty of Medicine, Mashhad University of Medical Sciences, Mashhad, Iran; ^11^Department of Hematology and Medical Oncology, Winship Cancer Institute, Emory University School of Medicine, Atlanta, GA 30322, USA; ^12^Applied Biomedical Research Center, Pharmaceutical Technology Institute, Mashhad University of Medical Sciences, Mashhad, Iran; ^13^Biotechnology Research Center, Pharmaceutical Technology Institute, Mashhad University of Medical Sciences, Mashhad, Iran; ^14^Department of Medical Biotechnology and Nanotechnology, Faculty of Medicine, Mashhad University of Medical Sciences, Mashhad, Iran; ^15^Department of Biotechnology, School of Pharmacy, Mashhad University of Medical Sciences, Mashhad, Iran

## Abstract

Cancer is a leading cause of death and disability worldwide. Epigenetic deregulation is one of the most critical mechanisms in carcinogenesis and can be classified into effects on DNA methylation and histone modification. MicroRNAs are small noncoding RNAs involved in fine-tuning their target genes after transcription. Various microRNAs control the expression of histone modifiers and are involved in a variety of cancers. Therefore, overexpression or downregulation of microRNAs can alter cell fate and cause malignancies. In this review, we discuss the role of microRNAs in regulating the histone modification machinery in various cancers, with a focus on the histone-modifying enzymes such as acetylases, deacetylases, methyltransferases, demethylases, kinases, phosphatases, desumoylases, ubiquitinases, and deubiquitinases. Understanding of microRNA-related aberrations underlying histone modifiers in pathogenesis of different cancers can help identify novel therapeutic targets or early detection approaches that allow better management of patients or monitoring of treatment response.

## 1. Introduction

Epigenetics is defined as stable and heritable alterations in gene expression and cellular function without changes to the original DNA sequence and can still be passed on from generation to generation [1]. This term was first used to describe gene-environment interactions that lead to manifestations of various phenotypes during development. Epigenetic changes are perceived to be key contributors to cell differentiation and acquisition of different cell fates with the background of the same genome. DNA methylation, histone modification, and RNA modifications are crucial components of epigenetics [2–4].

DNA methylation is defined as the addition of methyl groups (CH_3_) on cytosine residues of CpG islands, especially those located in the gene promoter region [2, 5]. For instance, the promoter regions of microRNA (miR) 101-2, miR-126, miR-148a, miR-152, and miR-185-5p are hypomethylated in prostate cancer [6], and the promoter region of miR-200b is hypermethylated in the metastatic phase of the same disease, leading to increased and decreased gene expression [7].

Histone modification is a post-translational rearrangement of histone proteins driven by methylation, acetylation, phosphorylation, ubiquitylation, and sumoylation [8]. RNA modification and noncoding RNA-associated gene silencing are involved in epigenetics by DNA methylation and/or histone modifications in two ways: (1) changing the structure of RNA and interference with RNA and proteins interactions and (2) inciting subsequent events by altered RNA-binding proteins (RBPs) [4].

These regulatory mechanisms can cause gene expression to be turned on or off [8]. Several studies have suggested that such modifications may play a role in inducing various disorders, most notably cancer [9]. Aberrant regulation of proteins or enzymes that regulate these modifications is a common feature in most cancers [9]. Among the multiple gene expression events regulated by these enzymes, the most pivotal genes controlling the cell cycle are refined into oncogenes or tumor suppressors [10].

MicroRNAs (miRNAs and miRs) are small noncoding RNAs with a length of approximately 22 nucleotides [11]. In the nucleus, they are first transcribed as 70 base-pair long double-stranded pri-miRNAs containing stem-loop structures [11]. Then, they undergo a cascade of processes in which they are converted to pre-miRNAs and transmitted to the cytoplasm, where they are cleaved to form single-stranded mature miRNAs [11]. With the cooperation of the RNA-induced silencing complex (RISC), mature miRNAs can seek and bind to complementary sequences in the target mRNA [11]. Depending on the exact or imprecise miRNA-mRNA interactions, mRNA eradication or translational hindrance will occur [11].

Cancer development is a multistep process, and genetic alterations in every step are manifested by significant dysregulation of proteins involved in cell cycle regulation, which may have been triggered by miRNAs [11, 12]. miRNAs can accomplish this by not only directly blocking the expression of the targeted genes but also by regulating the expression of epigenetic modifiers as well as histone methylases that play role in chromosomal structural changes [12, 13]. Hence, microRNAs can also participate in regulating epigenetic mechanisms in cancer, and their abnormal expression profiles have been frequently indicated in malignancies.

This review will discuss histone modifications and the microRNA-mediated regulation of the histone modification machinery in cancer.

## 2. Alterations of Histone Modification in Cancer

Histones are lysine–arginine abundant proteins involved in chromosome condensation, consisting of four core types (H2A, H2B, H3, and H4) located in the bead of the nucleosome, along with two linker histones (H1/H5). The amino and carboxy termini of these proteins may undergo modifications, such as methylation, acetylation, phosphorylation, sumoylation, ubiquitination, and ADP-ribosylation, which are pivotal for transcriptional regulation. The addition of acetyl group on lysine residues of the H3 and H4 classes of histones results in a lightly packed chromosome structure and transcription activation [14]. Lysine residues can be mono-, di-, or trimethylated although arginine residues can only be mono- or dimethylated. Functioning like a double-edged sword, histone methylation may lead to the development of either heterochromatin or euchromatin structures depending on factors such as the number of methyl groups, type of histone, and amino acid residues [15]. The heterochromatin formation that is followed by H3 three methylation at lysine 9 (i.e., H3K9me3) inclines towards transcriptional inactivation. However, histone H3 Lys-4 methylation (i.e., H3K4me) may lead to a transcriptional activation change [14]. Moreover, phosphorylation that occurs on serine residues, especially on H1 and H3 histones, can lead to gene expression induction. Ubiquitination usually takes place on H2A and H2B proteins and is correlated with activation although ADP-ribosylation is associated with chromosomal condensation [16]. The outcome of the ubiquitin-like modifier (SUMO) has been termed histone sumoylation and is related to transcription inactivation.  Table 1 summarizes the above effects of different histone modifications and their impact on transcriptional regulation.

Given that histone modification affects gene transcription and appears early in tumorigenesis, considerable research has been carried out on the role of these alterations in malignancies. H4K16 hypoacetylation has been identified in breast, colon, lung, and liver cancers as well as in medulloblastomas [17, 18]. This abatement in acetylation, together with H4K20me3 loss and co-occurrence in repetitive sequences with decreased DNA methylation and H4k20 trimethylation, has been reported in many cancers such as breast and lung cancer [17, 18]. Besides lysine 20 and 16 H4, alterations of other lysine residues of this histone are prominent in malignancies. For instance, H4k5 and H4K8 hyperacetylation along with H4K12 deacetylation has been demonstrated in lung cancer [15]. As stated in a meta-analysis study, elevated levels of H3K4me3 and decreased levels of H3K4me2 are indicators of a poor outcome in cancer patients [19]. Other lysine residues of the H3 shift have been found to be elevated in tumorigenesis. For example, loss of H3K4me2, H3K18ac, H3K9ac, and H3K9me3 is related to lung cancer recurrence and poor prognosis [15]. These modifications are located in promoters of transcribed genes and are essential for transcriptional suppression [20]. Moreover, H3K9me3 could serve as prognostic and staging estimation biomarkers in gastric cancer [21]. Loss of H2Bub1 is found in the development of numerous cancers as well as breast, lung, and colorectal cancer [22]. In addition, extensive phosphorylation of H2Bser32 has been shown in skin cancer cells [23].

All of these modifications are involved in malignancy induction by revising tumor suppressors or oncogene expression. H3 and H4 hypoacetylation and hypermethylation lead to p21WAF1 tumor suppressor inactivation [17]. Loss of H3K9ac, H3K4me2, and H3K9me3 has been linked to an increase in oncogene levels, such as MEIS1 and HOXA9 [21]. Increased and decreased methylation of H3K9 and H3K4, respectively, have been associated with p16INKA and p14ARF tumor suppressor gene reductions in some neoplasia [21].

Furthermore, dysregulation of histone-modifying enzymes in cancer and their distinct expression profile in tumor cells compared to normal cells have been identified in some studies [24]. For example, histone deacetylase (HDAC) reduction may be correlated with lung cancer promotion. However, HDAC overexpression in colon, prostate, and gastric cancers can result in blockage of tumor suppressor genes [15, 25]. EZH2, an H3K27 trimethylation enzyme, raises in breast carcinoma [15]. A histone methyltransferase upregulation along with histone demethylase downregulation has been found in prostate tissues. Moreover, HDM (histone demethylase) high expression levels have been reported in the liver, while HDM and HMT are largely decreased in the brain. By contrast, elevated levels of HDMs have been found in prostate and brain malignant cells [26].

## 3. Regulation of miRNA Expression by Histone Modifiers

miRNAs are involved in the regulation of biological processes such as development, growth, differentiation, proliferation, and apoptosis [27]. Alterations in miRNA function have been reported in all diseases and conditions, markedly in cancer [27]. Furthermore, miRNA expression profiling can provide prognostic and treatment monitoring biomarkers, as well as indicators of recurrence [27]. They can target cellular checkpoint genes and genes involved in mitosis division and apoptosis. For this reason, they are typically divided into two classes, onco MIRs and tumor suppressor MIRs [28].

### 3.1. Onco MIRs

Onco MiRs (oncomir) switch tumor-linked operations, such as unlimited cell growth, transformation, and metastasis. miR-21 is an oncomir known to be elevated in many cancers and drives cell proliferation. A sort of H3K4 demethylase, known as RBP2, can decrease miR-21 levels followed by decreased H3K4 trimethylation of its promoter and could act as a novel treatment in chronic myeloid leukemia cells [29]. Overexpression of miR-224 oncomir is regulated by H3K9 and H3K14 acetylation in hepatocellular carcinoma [30]. The miR-155 promoter will alter heterochromatin as a result of H2A and H3 deacetylation carried out by BRCA1 and HDAC2, resulting in increased cell growth and proliferation [31].

### 3.2. Tumor Suppressor miRs

miR-29 is known as a tumor suppressor gene because of its function in preventing cell growth and proliferation. H3K27 trimethylation is accomplished by recruiting YY1 and Ezh2. This change is related to the miR-29 promoter and could repress its expression in skeletal muscle cells. Aberrant downregulation of miR-29 by raised H3K27me3 is found in rhabdomyosarcoma [32]. Furthermore, induction of H3K27me3 and histone acetylation results in reduced levels of miR-31 [20]. miR-34a, a tumor suppressor gene governing apoptosis and cell cycle obstruction, can be arrested due to H3K27 trimethylation of its promoter by EZH2 in pancreatic ductal adenocarcinoma [33]. miR-125b has a tumor suppressor function in breast cancer, and its expression can be silenced through H3K9 and H3K27 trimethylation in breast cancer cell lines since miR-125b-1 promoter is located in a CpG island [34]. miR-30 family members classified as tumor suppressors are downregulated in a variety of malignancies. In esophageal squamous cell carcinoma cells, miR-30c expression is inactivated as a result of H2B and H4 histone deacetylation at the promoter region of miR-30c [35]. Downregulation of miR-15a, miR-16, and miR-29b in chronic lymphocytic leukemia is mediated by HDACs [36]. Other examples of these miRNAs are given in Table 2.

## 4. Epi-miRNAs and Histone Modification Machinery in Cancer

The epigenetic profile in numerous cancers is altered by the action of miRNAs [49, 50]. These small noncoding RNAs can target different enzymes involved in histone modifications. In the following sections, we discuss these miRNAs in more detail according to their enzyme targets.

### 4.1. miRNA-Mediated Regulation of Histone Acetyltransferases

There are numerous studies demonstrating miRNA-mediated regulation of different histone acetyltransferases including EP300, PCAF, TIP60, and hCLOCK (Table 3). The polycistronic miR-106b-25 cluster, which consists of miR-106b, miR-93, and miR-25, is associated with proto-oncogenic functions and uncontrolled growth. As Zhou et al. suggested in their research, this oncoMIR cluster can target EP300, a histone acetyltransferase that modifies chromatin remodeling, cellular growth, and differentiation processes. Upregulation of the miR-106b-25 cluster in breast cancer leads to metastatic alterations by EP300 blockage [51]. PCAF or p300/CBP-associated factor is another histone acetyltransferase upregulated in prostate cancer cells, and its expression level is decreased through miR-17-5p binding [52]. miR-22, elevated in most cancers, can target and repress a lysine acetyltransferase named TIP60 [53]. Accordingly, miR-22 suppression may influence treatment in breast cancer cell lines by reducing metastasis [53]. hCLOCK, a target of miR-124, is significantly decreased in response to the elevated levels of miR-124 in a human colorectal cancer cell line known as LOVO cells [54].

### 4.2. miRNA-Mediated Regulation of Histone Deacetylases

HDACs are classified into four classes including classes I–IV [55]. There has been accumulating evidence of miRNA-mediated regulation of these enzymes (Table 4), and the following section describes these according to their classifications.

#### 4.2.1. Class I Rpd3-Like Proteins (HDAC1, HDAC2, HDAC3, and HDAC8)

The upregulation of HDAC1 results in uncontrolled growth and cisplatin-resistant in ovarian cancer cells. miR-34a suppresses this process by targeting HDAC1 [56]. Moreover, an HDAC1 decrease through the action of miR-34a can overcome treatment resistance in breast cancer [67]. miR-145 is a tumor suppressor, and its inactivation can increase HDAC2 levels and lead to the development of hepatocellular carcinoma [57]. Kim et al. demonstrated that miR-326 inhibition may give rise to HDAC3 upregulation followed by a response to anticancer drugs in hepatoma and melanoma-related cell lines [58]. As demonstrated by Wang et al., the expression of miR-216b, which controls HDAC8, is downregulated in gastric adenocarcinoma [68].

#### 4.2.2. Class II Hda1-Like Proteins (HDAC4, HDAC5, HDAC6, HDAC7, and HDAC9)

Alterations in HDAC4 expression occur via several miRNAs and vary based on cancer type. Amodio et al. found that miR-29b/HDAC4 serves as an epigenetic loop in multiple myeloma and the induction of miR-29b expression could repress HDAC4 and result in cell survival and reduced malignancy in myeloma [59]. However, Ahmad et al. reported that induction in miR-10b levels leads to the tamoxifen-resistance phenotype due to loss of HDAC4 expression in ER-positive breast cancer cells [60]. Moreover, in another study carried out on fulvestrant-resistant breast cancer cells, it was found that miR-22 overexpression inhibits HDAC4 and promotes cell proliferation [61]. One observation has already drawn attention to the paradox in HDAC4 function in breast tumorigenesis since its suppression through miR-125a-5p inhibits tumor progression [62]. The downregulation of this miRNA, which targets HDAC5 in breast cancer cells, is related to the blockade of cancer progression [63]. Liu et al. found that the expression level of HDAC5 was reduced by binding of miR-589-5p to the 3' untranslated region (UTR) of HDAC5 in non-small-cell lung cancer (NSCLC) cells [64]. HDAC6 is downregulated via miR-221, which is upregulated in liver cancer cells [65]. Diminished levels of miR-489 induce metastatic processes of gastric cancer cells and block HDAC7 expression [66]. The levels of miR-34a may reflect the tumor-suppressive effect, and its transcription is abolished in cancer stem cells [67]. miR-34a targets HDAC7 and thereby contributes to the regulation of therapy resistance in breast cancer [67]. It has been shown that HDAC7-mediated deacetylation of a specific lysine residue of a heat shock protein, namely HSP70 K246, contributes to the augmentation of treatment resistance, in which miR-34a may exert a suppressive role through its downstream effectors HDAC7 [67]. miR-377 depression is found in oral squamous cell carcinoma, influencing the expression of HDAC9 and tumor invasion [69].

#### 4.2.3. Class III Sir2-Like Proteins (SIRT1, SIRT2, SIRT3, SIRT5, SIRT6, and SIRT7)

SIRT is a family of histone deacetylase compromised seven proteins and divided into four classes. Class I includes SIRT1, SIRT2, and SIRT3. Class II consists of SIRT4. SIRT5 belongs to class III, and SIRT6 and SIRT7 are class IV members [80]. Being elevated in breast cancer, SIRT1 plays a significant role in this disorder [62]. Several miRNAs interact with SIRT1. miR-34a, a direct regulator of SIRT1, is decreased in breast cancer [62]. As suggested by Li et al., induction of miR-34a along with 5-FU therapy has significant antitumor effects in breast malignancies [70]. In addition, Eades et al. demonstrated a diminished level of miR-34a and an increased level of SIRT1 in CD44+/CD24− breast cancer stem cells [71]. Moreover, SIRT1 forms a negative feedback loop with miR-200a, with overexpression of SIRT1 being related to decreased levels of miR-200a in patients suffering from breast cancer [72]. Reduced levels of miR‐590‐3p are associated with upregulation of SIRT1 followed by hyperacetylation of p53 and increased levels of BAX and p21, leading to malignant characteristics in breast cancer cells [73]. It has been shown that downregulation of miR-22 is accompanied by overexpression of SIRT1 in breast cancer tissues [74]. Downregulation of SIRT2 as a result of miR-150 augmentation has been observed in NSCLC cells, potentially serving as a survival element through blocking the AKT signaling pathway in this cell line [75]. SIRT3 is fine-tuned by miR-708-5p that is upregulated in pancreatic ductal adenocarcinoma [76]. Dang et al. confirmed that miR-299-3p, downregulated in hepatocellular carcinoma cells [77], exerts tumor-suppressive roles by modifying growth and metastasis through SIRT5 inactivation [77]. SIRT6 is a direct target of miR-186, and Ruan and colleagues demonstrated that miR-186 upregulation could serve as a treatment target in lung cancer [78]. miR-3666 inhibits breast cancer cell proliferation by targeting SIRT7. Also, overexpression of SIRT7 in response to downregulation of miR-3666 has been observed in NSCLC and breast cancer cells [79, 81].

### 4.3. miRNA-Mediated Regulation of Histone Methyltransferases

There are two major types of histone methyltranferases termed histone lysine N-methyltransferases and histone arginine N-methyltransferases. The following sections discuss the miRNA-mediated regulation of both types.

#### 4.3.1. miRNA-Mediated Regulation of Histone Lysine Methyltransferases

Lysine methyletransferases (KMTs) are divided into groups based on the site of methyl group addition [82]. In this section, we discuss miRNA-mediated regulation of histone lysine methyltransferases considering their classification. The data summary is given in Table 5.

*(1) Suv39H1, Suv39H2, SETDB1, and G9A/EHMT2 (H3K9)*. miR-125a-5p, a recognized prognostic factor in gastric cancer, regulates SUV39H1 (KMT1A) and prevents angiogenesis [83]. The downregulation of this miRNA has been correlated to SUV39H1 upregulation in hepatocellular carcinoma cells [84]. In addition, miR-122 is another regulator of SUV39H1 in hepatocellular carcinoma cells [85]. miR-675 along with PKM2 leads to decreased levels of SUV39H2 and cancer progression in liver stem cells via c-myc upregulation [86]. Low levels of miR-212 have been linked to lung cancer and can target G9a/KMT1C [87]. Wu et al. identified SETDB1 upregulation as a target of miR-381-3p in breast malignancies [88].

*(2) KMT2A (MLL1)*. KMT2A is a direct target of hsa-miR-22-3p, and hsa-miR-22-3p upregulation has been found in the metastatic form of prostatic cancer [89].

*(3) NSD1 and ASH1L (H3K36)*. miR-142 could inhibit ASH1L (KMT2H), and its downregulation leads to increased levels of ASH1L in leukemia [90]. Moreover, Colamaio et al. described the same finding in thyroid tumors [91]. KMT3B (NSD1) is one of the miR-181a targets, and the oncogenic role of hsa-miR-181a suggests it could be a prognostic biomarker in endometrial cancer [92].

*(4) SMYD3 (H4K5)*. miR‐124 acts as a SMYD3 (KMT3E) expression modifier, and its decreased levels that result in cellular invasive criteria have been shown in intrahepatic cholangiocarcinoma cells [93]. miR-346 downregulation induces SMYD3 upregulation in hepatocellular carcinoma and could be a poor prognostic factor [94].

*(5) hDOT1L (H3K79)*. DOT1L (KMT4) is blocked by miR-133b. miR-133b is a tumor suppressor, and low levels cause chemoresistance in colorectal cancers [95].

*(6) SET8 (H4K20)*. SET8 is a histone methyltransferase that adds one methyl group on lysine 20 of H4. Its expression can be switched by miR-502, and it has oncogenic effects and contributes to cell growth and migration. This oncogene expression can be increased due to reduced levels of miR-502 in many malignancies including breast cancer, ovarian cancer, small-cell lung cancer, colorectal cancer, non-Hodgkin's lymphoma, esophageal squamous cell carcinoma, clear cell renal cell carcinoma, and hepatocellular carcinoma [96, 98, 102, 103]. For these reasons, it has been considered as a potential target for cancer therapy. Also, a single nucleotide polymorphism (SNP; rs16917496) located at the binding sequences of miR-502 may affect SET8 expression and has been associated with the risk of these malignancies [97, 99–101, 104, 124]. Moreover, this histone methyltransferase could be regulated with miR-7, and its suppression prevents cell invasion in breast cancer [105].

*(7) EZH1 and EZH2 (H3K27)*. Elevation of KMT6B (EZH1) caused by miR-17-5p downregulation is related to erlotinib resistance in NSCLC [106]. miR-765 is another regulating element of EZH1, and its downregulation is associated with aggravation of breast cancer [107]. Additionally, a study by Hu J. et al. showed that miR-93 is a key regulator of EZH1 as well as JAK1, STAT3, AKT3, SOX4, and HMGA2 in breast cancer stem cells [108]. In a study of prostate cancer stem cells, Lai et al. reported miR-574-5p depletion along with EZH1 and REL increased levels [109].

Multiple microRNAs can alter EZH2 expression. Effects of miR-101 on EZH2 have been observed in various cancers [125], and downregulation of this microRNA and elevation of EZH2 have been found in NSCLC tumor tissues, prostate cancer, and renal cancer [110–112]. According to a study by Konno et al., the microRNA-101-EZH2/MCL-1/FOS axis may be a target for endometrial cancer treatment [113]. miR-26a as an apoptosis inducer is another regulator of EZH2, and its diminished levels of this miRNA have been found in lung cancer, rhabdomyosarcoma, and prostate cancer [114, 116, 117]. It plays a role in Burkitt lymphoma and prostate cancer prevention by targeting c-myc, a transcription factor of EZH2 [115, 117], and is involved in nasopharyngeal carcinoma inhibition by mitigating cyclin D3, E2, CDK4, and CDK6 along with overexpression of the CDK inhibitors p14ARF and p21CIP1 [118]. miR-137 downregulation is related to EZH2 upregulation in hepatocellular cell carcinoma and miR-137 inhibits hepatocellular cell carcinoma progression by EZH2 suppression [119]. In addition, miR‐124 manifests the same action on EZH2 in cancer cell lines [120]. Liu and co-workers demonstrated miR-138 deficiency can give rise to cellular metastasis via targeting VIM, ZEB2, and EZH2 in squamous cell carcinoma cell lines [121]. A study by Koumangoye et al. implicated EZH2 and HDAC3 as indirect targets of miR-31 in esophageal cancer cells as their upregulation was associated with raised levels of miR-31 by directly regulating SOX4, which then acts on EZH2 and HDAC3 [125]. miR-98 is a key regulator of EZH2 and, according to the investigation of Liu et al., treatment of ovarian cancer stem cells with an expression plasmid containing EZH2-targeted miR-98 blocked cell growth, and ameliorated cell cycle status via increasing p21CIPI/WAF1 and E2F1 tumor suppressors and downregulating c-Myc and CDK2/cyclin E complex proto-oncogenes [126].

#### 4.3.2. miRNA-Mediated Regulation of Histone Arginine Methyltransferases

Protein arginine methyltransferases (PRMTs) are involved in histone post-translational methylation [126], and multiple investigations have found miRNA-mediated regulation of these enzymes in various cancers (Table 6). For example, an investigation by Li et al. found that diminished miR-503 levels were associated with PRMT1 elevation in hepatocellular carcinoma [127]. CARM1 elevation improves cell growth in colorectal malignancies. miR-195 is an antitumor element and increases radiosensitivity in colorectal cancer cells as a result of PRMT4/CARM1 targeting [128, 129]. miR-424-5p is another regulator of CARM1. Wang and his colleagues indicated its low levels in NSCLC tissues. Also, they stated that high levels of miR-424-5p could suppress CARM1 followed by decreasing tumor development [130]. Upregulation of PRMT5 is related to the impaired expression of miR‐92b and miR‐96 in lymphoid cancer cell lines and leads to ST7 inactivation by H3R8 and H4R3 methylation [131]. miR-1266 overexpression appears to decrease PRMT5 levels and modify cellular growth and proliferation, thus suggesting its use as a novel therapeutic target in prostate cancer [132]. PRMT9 expression is shifted by direct binding of miR-543 to its 3ʹ-UTR and increased levels of this miRNA have been associated with downregulation of PRMT9 and osteosarcoma cell growth [133].

### 4.4. miRNA-Mediated Regulation of Histone Demethylases

There are multiple histone demethylase enzymes divided into lysine and arginine methyltransferase groups. Histone lysine demethylases are classified into KDM1-8 families [134]. miRNA-mediated regulation of histone demethylases in cancer is described in the following sections and summaries shown in Table 7.

#### 4.4.1. KDM1 (LSD1)

LSD1 is a histone demethylase, controlled by miR-137 in a negative feedback loop in endometrial cancer [135]. miR-302 has been shown to be downregulated in hepatocellular carcinoma cells, and this miRNA could decrease LSD1 (AOF2) levels resulting in drug sensitivity improvement and c-myc suppression [136]. By contrast, Bourguignon et al. reported miR-302a and miR-302b upregulation and AOF2 suppression in mouse tumors and human head and neck squamous cell carcinomas [137]. Likewise, miR-302a overexpression along with AOF2, BCRP, and permeability glycoprotein 1 decrease has been found in prostate cancer [138].

#### 4.4.2. KDM2 (FBXL10 and KDM2B)

FBXL10 is a direct target of miR-146b, and downregulation of this miRNA in later stages of epithelial ovarian cancer has been linked to FBXL10 increase that, in turn, induces metastasis. However, in the early stage of the disease, miR-146b reduction results in overexpression of cyclin D1 and cell proliferation [139]. Hong and co-authors reported miR-448 overexpression in gastric cancer. This miRNA binds to KDM2B and suppresses its expression that results in myc induction [140]. KDM2B is upregulated in cervical cancer cell lines and downregulates miR-146a-5p expression that, in turn, increases c-myc levels [141].

#### 4.4.3. KDM3 (JMJD1A)

JMJD1A is a direct target of miR-627, and its low expression is related to growth and differentiation inhibition [142]. LMP1 and LMP2A induce miR-155 upregulation followed by JMJD1A suppression in nasopharyngeal carcinoma cases with poor prognosis [143]. There is a loop between JMJD1A, EZH2, and let-7c in NSCLC cells, and inhibition of JMJD1A can downregulate EZH2. EZH2 depletion causes the let-7c increase. However, let-7c binds to the 3'-UTR of JMJD1A and EZH2 and shifts their expression in a feedback loop, predisposing them to cancer phenotype amelioration [144]. EWS/Fli1 oncoprotein participates in miR-22 suppression that can regulate KDM3A (JMJD1A/JHDM2A) expression. Downregulation of KDM3A results in tumorigenic profile reduction in Ewing sarcoma [145].

#### 4.4.4. KDM4 (JMJD2B and JMJD2C)

JMJD2B can be regulated by miR-491-5p, and its overexpression via miR-491-5p downregulation has been observed in ER*α*-positive breast cancers and cell lines. miR-491-5p upregulation results in cell cycle arrest and attenuates growth through inhibition of JMJD2B in the same cancer [146]. miR-491-5p carries out the same function in gastric cancer by binding to the 3'UTR of JMJD2B. This miRNA is a tumor suppressor, and its downregulation has been used as a biomarker in this disease [147]. Yong et al. illustrated that circZMYM2 inhibits miR-335-5p expression in prostate cancer cells and tissues and induces prostate cancer development. Overexpression of miR-335-5p as a treatment can block JMJD2C expression and reduce prostate cancer progression [148].

#### 4.4.5. KDM5 (JARID1B and RBP2)

Studies have shown that JARID1B levels are ameliorated by miR‐137 [152]. Thus, increased levels of JARID1B and reduced levels of miR‐137 have been found in acute lymphoblastic leukemia cell lines, and increased miR-137 can prevent uncontrolled cell proliferation [152]. miR-363-3p is expressed as a result of HIF-2a induction, followed by p21 suppression and resulting in JARID1B elevation in melanoma [151]. JARID1B/KDM5B is also known to be regulated by miR-138, miR-381-3p, and miR-486-5p as observed in breast cancer [153]. Reference [154] RBP2 is a direct target of miR‐212, and its overexpression in response to downregulation of miR‐212 has been found in gastric carcinogenesis and hepatocellular carcinoma [149, 150]. miR-212 acts as a tumor suppressor, and its aberrant expression participates in the augmentation of a cell proliferation phenotype through induction of impaired RBP2 expression and downregulation of P21CIP1/P27kip1 [149, 150].

#### 4.4.6. KDM7 (PHF2)

miR-221 can target PHF2, and its upregulation along with PHF2 decrease has been shown in hepatocellular carcinoma cells [155].

#### 4.4.7. JMJD6 (Arginine Demethylase)

JMJD6 is a miR-770 direct target, and its overexpression due to miR-770 downregulation followed by WNT/*β*-catenin pathway activation has been found in NSCLC [156]. Furthermore, miR-146a and miR-193a target JMJD6 overexpression has been described in melanoma progression [157].

### 4.5. miRNA-Mediated Regulation of Histone Kinases

There are several clusters of kinases classified by kinase domain sequences similarity, biological function, and other criteria [158]. miRNA-mediated regulation of histone kinases is described below and summarized in Table 8.

#### 4.5.1. AGC (RPS6KA4/MSK2, PRKCD, PRKCB, RSK2, and PRKDC)

Reduction of miR-517a is found in bladder cancer cell lines. It has a tumor-suppressive effect and can target RPS6KA4/MSK2 [159]. The PRKCD kinase can be regulated by many kinds of miRNAs. miR-181a acts as an oncogene, and its upregulation decreases the chemosensitivity of cervical squamous cell carcinoma via PRKCD inhibition [160, 161]. In ovarian cancer tissues, miR-181c was found to be decreased and caused a PRKCD increase via binding to its 3'UTR [162]. As reported by Yao et al., PRKCD knockdown in A2780 cells resulted in cell cycle arrest in the G1 phase and metastasis inhibition [162]. However, in another study carried out by Zhao et al., miR-224 functioned as an oncogene targeting PRKCD and enhanced chemoresistance in ovarian papillary serous carcinoma [163]. The downregulation of miR-197-3p along with PRKCB overexpression in gastric cancer has been reported [164]. Low levels of miR-634 increase RSK2 in relation to cisplatin resistance in ovarian cancer [165]. miR‐488‐3p represses PRKDC and increases malignant melanoma cell sensitivity to cisplatin [166].

#### 4.5.2. Nonreceptor Tyrosine Kinase (JAK2)

JAK2 is one of the most important kinases involved in histone regulation and the key member of the JAK2/STAT3 signaling pathway recruited in inflammation and apoptosis [218]. The level of JAK2 is regulated by a variety of miRNAs. The miR-543 expression serves as a tumor suppressor and prevents cell proliferation by targeting JAK2 and STAT3 in hepatocellular carcinoma [167]. Low levels of miR-216a and high levels of JAK2 in pancreatic cancer were reported in two distinct studies [168, 169]. JAK2, BCl-2, and surviving are direct targets of miR-204 in breast cancer, and the opposite expression pattern of miR-204-JAK2 has been reported in NSCLC [170, 171]. Furthermore, miR-204 can be used as a prognostic marker in these cancers [170, 171]. Several studies reported the downregulation of miR-375 in gastric cancer and its function in inhibiting tumor development by suppressing JAK2 [172, 173]. miR-135a is another regulator of JAK2 in different cancers such as lymphoma and renal and gastric carcinoma and can influence apoptotic genes as well as Bcl-x and Bcl-2 [174–176].

#### 4.5.3. Tyrosine Kinase-Like (LIMK2)

LIMK2 plays an oncogenic role in bladder cancer and can be decreased by miR‐135a [183].

#### 4.5.4. Other Kinases Family (NEK6, AURKA, and BUB1)

miR-23 can target NEK6, the enzyme that negatively regulates p53. The natural substance berberine plays a role in hepatocellular carcinoma treatment by activating this signal [184]. In another study, miR-26 was documented as a modulator of NEK6 in Marek's disease lymphoma and suppressed cell proliferation [185]. NEK6 is an oncogene and a direct target of miR-506-3p in retinoblastoma [186]. miR-124-3p was found to be decreased in bladder cancer tissues and cell lines and glioblastoma, and its downregulation led to AURKA increase [187, 188]. In NSCLC, miR-32 targets AURKA and causes p53 inhibition [189]. miR-137 can suppress AURKA expression and prevent drug resistance in multiple myeloma [190]. let-7 is another modulator of AURKA that is downregulated in hepatocellular cancer [191]. Diminished levels of miR-490 have been found in hepatocellular carcinoma, and AURKA as an established target of miR-490 was elevated [192]. Gomaa and colleagues found a relationship between miR-4715-3p downregulation caused by methylation and AURKA overexpression in gastrointestinal cancers that were attenuated by 5-Aza-2'-deoxycytidine, a demethylation element [193]. Downregulation of miR-490-5p and miR-145-3p along with an increase in BUB1 was found in hepatocellular carcinoma and prostate cancer, respectively, leading to cancer cell invasion [194, 195].

*(1) CAMK (DAPK3, CHEK1, CHEK2, and AMPK)*. DAPK3 is a p53-activating kinase and a direct target of miR-1307, which is overexpressed in chemoresistant ovarian cancer cell lines [205]. CHEK2 can be regulated by miR-191 in osteosarcoma, and miR-191 provokes cell growth [196]. miR-182-5p overexpression promotes CHEK2 suppression and is involved in breast cancer [197]. CHEK1 is controlled by several miRNAs, and its alteration functions a double-edged sword in different malignancies. Downregulation of miR-195 in NSCLC is correlated with augmentation of CHEK1, which is an indication of poor survival [198]. However, downregulation of miR-195 in colon cancer cell lines can suppress the viability of cancer 5-FU-resistant cells by increasing CHEK1 and might be considered as a treatment in colon cancer [199]. miR-497 is another regulator of CHEK1 in hepatocellular carcinoma [200]. miR-145 and miR-424 downregulation together with CHEK1 upregulation were found in bladder cancer and cervical cancer, respectively [201, 202]. miR-15 can increase breast cancer cells irradiation by targeting CHEK1 [203]. p53 induces miR-16 and miR-26a expression, and they inhibit CHEK1 expression leading to enhanced apoptosis and better survival in breast and prostate cancers [204]. miR-451 overexpression hampers AMPK and promotes mTOR and FCN1 expression in colorectal cancers leading to proliferation induction [206]. A similar result was found with exogenous expression of miR-25-5p [207]. miR-101 and miR-34 are other regulators of AMPK, and an increase in their expression is associated with proliferation arrest and initiation of apoptosis in breast and prostate cancer, respectively [208, 209].

*(2) STE (STK4 and PAK2)*. miR-18a elevation motivates prostate cancer development through STK4 suppression [210]. PAK2 is modulated by various microRNAs in different cancers. miR-4779, miR-216a-5p, miR‐137, and miR‐7‐5p are tumor suppressors that regulate PAK2 in colon cancer, breast cancer, melanoma, and NSCLC. Downregulation of these miRNAs has been associated with tumor growth and proliferation [211–214]. Moreover, Shuang et al. showed that miR-134 suppression led to augmentation of PAK2 followed by paclitaxel resistance in human ovarian cancer cells [215]. In addition, CCHE1 inhibited miR‐922, which, in turn, leads to an incline in PAK2 and participates in tumorigenesis of oral squamous cell carcinoma development [216], and miR-26a halted cancer invasion by restricting PAK2 in hepatocellular carcinomas [217].

*(3) CMGC (MAP3K8, GSK3B, and CDK3)*. miR-589-5p limits MAP3K8 expression and causes suppression of CD90+ cancer stem cells in hepatocellular carcinoma [179]. miR-144-3p and miR-509-3p participate in the inhibition of cancer cell proliferation by inactivating MAP3K8 in renal cell carcinoma [180, 181]. miR-769 promotes melanoma improvement by modulating GSK3B expression [182]. CDK3 is also modulated by numerous miRNAs. miR-873 controls CDK3 activity, and decreased levels of this miRNA have been described in breast cancer [177]. miR-873 shifts tamoxifen resistance by targeting CDK3 and inducing ER phosphorylation [219]. miR-4469 is another noncoding RNA that targets CDK3 elevation in primary breast tumors compared to metastatic ones [178].

### 4.6. miRNA-Mediated Regulation of Histone Phosphatases

The main groups of protein phosphatases are sorted considering the structural fold of the catalytic domain [220], and miRNA-mediated regulations of these are summarized in Table 9.

#### 4.6.1. PPM (PPM1D)

PPM1D has been found to be modulated by miR-499a-5p and miR-499a-5p downregulation followed by PPM1D upregulation in osteosarcoma [221].

#### 4.6.2. PPPL (PPP2CA and PPP2CB)

PPP2CA is a direct target of miR‐155, and its overexpression leads to PPP2CA low levels in colon cancer [222]. miR-650 acts as an oncogene, and its upregulation causes a PPP2CA decrease in thyroid cancer [223]. miR-130b overexpression provides invasion through PPP2CA targeting in glioma [224]. Upregulation of miR-1246 induces inflammatory element expression by targeting PPP2CB in breast cancer [225]. However, decreased levels of miR-129-5P have been found in papillary thyroid carcinoma, and PPP2CB expression was induced as its target [226].

#### 4.6.3. HAD (EYA1 and EYA2)

The apoptosis activator miR-101 can repress EYA1 and is diminished in breast cancer [227]. miR-562 is another modulator of EYA1, and its downregulation has been found in sporadic Wilms' tumor [228]. miR-338 and miR-30a are EYA2 regulators, and their downregulation increases EYA2 expression in breast cancer, and its reduction and epidermal growth factor receptor (EGFR) downregulation have been associated with lung metastasis [229, 230]. Furthermore, miR-30a overexpression was found to reduce EYA2 and attenuate cell metastasis in lung adenocarcinoma [231]. The expression of miR-219a-5p and miR-338-3p were shown to be diminished in osteosarcoma and cervical cancer, respectively, both targeting EYA2 [232, 233]. EYA3, a direct target of miR-708, was upregulated in Ewing sarcoma, promoting chemoresistance [234].

#### 4.6.4. CC1 (DUSP1)

DUSP1 is controlled by various miRNAs. In osteosarcoma, miR-34a targets DUSP1, and its repression is related to elevated levels of Bax and E-cadherin, along with diminished levels of Bcl-2, cyclin E, cyclin D1, and *β*-catenin [235]. Upregulation of miR-202-3p downregulates DUSP1 and provokes tumor development in gastric neuroendocrine neoplasms [236]. Various studies revealed that miR-324 and miR-101 function in DUSP1 targeting in hepatocellular carcinoma, and their upregulation impedes metastasis and promotes apoptosis, suggesting these as potential novel treatment targets [237, 238].

### 4.7. miRNA-Mediated Regulation of Histone Desumoylation

SENP1 is one of the most important desumoylation proteins modulated by various miRNAs (Table 10). SENP1 can be inactivated by multiple miRNAs. In lung cancer samples and cell lines, reduction of miR-138 along with SENP1 increase has been identified. miR-138 overexpression can elevate radiosensitivity by SENP1 blockage leading to increased apoptosis [239]. miR-133-3p and miR-186 are the regulators of SENP1 in colorectal and renal cell carcinoma, respectively. Their downregulation raises expression of the SENP1 oncogene in these cancers followed by accelerated proliferation [240, 241]. In prostate cancer, miR-145 loses its tumor suppressor activity, and this leads to increased SENP1expression. In addition, CDX2 can target miR-145-5p and decrease metastasis [242, 243].

### 4.8. miRNA-Mediated Regulation of Histone Ubiquitinations

RBX1, RNF8, HUWE1, and UHRF1 are histone ubiquitinating enzymes involved in different malignancies. Below, we summarize the miRNA-mediated effects on the regulation of these enzymes (Table 11). RBX1 has been shown to be decreased by miR-378 and miR-194 in lung and gastric cancer, and this suppresses proliferation and metastasis [244, 245]. Cheng et al. revealed that miR-542-5p targets HUWE1 and impedes osteosarcoma development [246]. RNF8 is another histone lysine ubiquitinase. miR-214 upregulation has been associated with RNF8 downregulation and provokes chromosomal instability in ovarian cancer cells [247]. Overexpression of miR-214 and miR-622 in breast cancer prevents cell proliferation by targeting RNF8 [248, 249]. Several miRNAs can control UHRF1 expression in different neoplasia. For example, low levels of miR-145 and miR-124 have been linked to increased levels of UHRF1 in bladder cancer cell lines and accelerated cell aggressiveness [201, 250, 251]. miR-9 and miR-202 serve as tumor suppressors and attenuate cell migration through UHRF1 blockage [252, 253]. Moreover, Goto et al. demonstrated the antitumor function of miR-101 in renal cell carcinoma through the inactivation of UHRF1 [254].

### 4.9. miRNA-Mediated Regulation of Histone Deubiquitination

USP3, USP7, and USP22 are three deubiquitination enzymes studied in cancers, and these are controlled by various miRNAs (Table 12). USP3 that is targeted by miR-224 is reduced in colorectal cancer and induces its progression [255]. Conversely, USP3 overexpression and miR-224 underexpression lead to an increase in the proliferation of gastric cancer cells [256]. Two separate investigations demonstrated that USP7 serves as a tumor suppressor, and an increase in miR-205 and miR-34a causes an alleviation in hepatocellular carcinoma [257, 258]. miR-30-5p may represent a novel therapeutic target in NSCLC, colorectal cancer, and nasopharyngeal carcinoma since it can mitigate tumorigenesis through USP22 suppression, leading to Wnt/*β*‐catenin signaling target genes (Axin2 and MYC) and Sirt1/JAK/STAT3 signaling modulation [259–261]. miR-29c enhances the chemosensitivity of pancreatic cancer cells by inhibition of USP22 (264). LncRNA HULC attenuates miR-6825-5p, miR-6845-5p, and miR-6886-3p levels that target USP22 in hepatocellular carcinoma [263]. In gastric cancer, POU2F1 can target miR-4490 and increase USP22 levels [264]. Additionally, miR-101 overexpression leads to USP22 depletion and reduces tumor progression in papillary thyroid carcinoma [265].

## 5. Conclusions and Future Prospects

With due attention to the high cancer mortality rate, early diagnosis and initiation of appropriate therapeutics are urgently needed. To further these objectives, it is crucial to increase our understanding of the mechanisms and pathways involved in malignancy progression and improvement. The histone-modifying enzymes that catalyze the remodeling of chromatin structures play a major role in cancer biology. In this review, we discussed miRNAs that interact with a complex array of histone modifiers and reviewed the effects of their aberrant expression in various cancers. These alterations impact the fluctuation of multiple cancer cell properties such as drug sensitivity, drug resistance, proliferation, apoptosis, and malignancy trajectories. Hence, recognition of these small noncoding RNAs is imperative for the early diagnosis of cancer and may lead to the identification of new biomarker tests to facilitate earlier diagnosis and treatment than is currently possible for the best outcomes.

## Figures and Tables

**Table 1:** Modified residues of histones, different histone modifications, and their impact on transcriptional regulation.

Modified residues of histones	Histone modification	Transcription regulation
Lysine	Acetylation	Activation
Lysine me1, me2, and me3	Methylation (lysine)	Repression/activation
Arginine me1, arginine me2a, and arginine me2s	Methylation (arginine)	Repression/activation
Serine, threonine, and tyrosine	Phosphorylation	Activation
Lysine	Ubiquitination	Activation
Glutamic	ADP-ribosylation	Repression
Lysine	Sumoylation	Repression

**Table 2:** Regulation of miRNA expression by histone modifiers.

miRNAs	Cancer type	Decrease/increase	Type of histone modification	Reference
miR-127	Primary human tumors	Decrease	Increased histone H3 acetylation and H3-K4 methylation	[37]
miRNA-1260b	Prostate cancer cells	Increase	Increased H3K9-me2, H3K9me3 and H3K27me3	[38]
miR-124a	Acute lymphoblastic leukemia	Decrease	Decreased levels of 3mk4H3 and AcH3 and increased levels of 2mK9H3, 3mK9H3, and 3mK27H3	[39]
let-7e, miR-1246, miR-1826, and miR-361-5p	Breast cancer	Decrease	Decreased H3K4me3	[40]
miR-615	Prostate cancer	Increase	Increased H3K9 acetylation	[41]
miR-29	B-cell lymphomas	Decrease	Increased histone deacetylation and trimethylation	[42]
miR-15a, miR-16, and miR-29b	Chronic lymphocytic leukemia	Decrease	Increased histone deacetylation	[36]
miR-15a and miR-16	Non-Hodgkin B-cell lymphomas	Decrease	Increased histone deacetylation (HDAC3)	[43]
miR-31	Breast cancer	Decrease	Increased methylation	[44]
miR-31	Adult T-cell leukemia	Decrease	Increased H3K9 and H3K27 methylation	[45]
miR-23a	Human leukemic Jurkat cells	Decrease	Increased deacetylation (HDAC4)	[46]
miR-139-5p, miR-125b, miR-101, let-7c, and miR-200b	Hepatocellular carcinoma	Decrease	Increased histone H3 lysine 27 (H3K27) trimethylating (EZH2)	[47]
miR-449	Hepatocellular carcinoma	Decrease	Increased histone deacetylation (HDAC1–3)	[48]
miR-224	Hepatocellular carcinoma	Increase	Increased histone acetyltation (Ep300)	[30]
miR-155	Breast cancer	Increase	Decreased H2A and H3 deacetylation (HDAC)	[31]

**Table 3:** miRNA-mediated regulation of histone acetyltransferases.

Target gene	miRNAs	Cancer tissue or cell line	Reference
EP300	miR-106b, miR-93, and miR-25	Breast cancer	[51]
PCAF	miR-17-5p	Prostate cancer	[52]
TIP60	miR-22	Breast cancer cell lines	[53]
hCLOCK	miR-124	Colorectal cancer cell line	[54]

**Table 4:** miRNA-mediated regulation of histone deacetylases.

Classification	Target gene	miRNAs	Cancer tissue or cancer cell line	Reference
Class I Rpd3-like proteins	HDAC1	miR-34a	Ovarian cancer	[56]
	HDAC2	miR-145	Liver cancer	[57]
	HDAC3	miR-326	Hepatoma and melanoma-related cell lines	[58]
Class II Hda1-like proteins	HDAC4	miR-29b	Multiple myeloma	[59]
	HDAC4	miR-10b	Breast cancer cells	[60]
	HDAC4	miR-22	Breast cancer cells	[61]
	HDAC4	miR-125a-5p	Breast cancer	[62]
	HDAC5	miR-125a-5p	Breast cancer	[63]
	HDAC5	miR-589-5p	Non-small-cell lung cancer cells	[64]
	HDAC6	miR-221 and miR-221	Liver cancer cells	[65]
	HDAC7	miR-489	Gastric cancer cells	[66]
	HDAC7	miR-34a	Breast cancer	[67]
	HDAC8	miR-216b	Gastric adenocarcinoma	[68]
	HDAC9	miR-377	Oral squamous cell carcinoma	[69]
Class III Sir2-like proteins	SIRT1	miR-34a	Breast cancer	[70]
	SIRT1	miR-34a	Breast cancer stem cell	[71]
	SIRT1	miR-200a	Breast cancer	[72]
	SIRT1	miR‐590‐3p	Breast cancer cells	[73]
	SIRT1	miR-22	Breast cancer tissues	[74]
	SIRT2	miR-150	Non-small-cell lung cancer (NSCLC) cells	[75]
	SIRT3	miR-708-5p and miR-708-5p	Pancreatic ductal adenocarcinoma	[76]
	SIRT5	miR-299-3p	Hepatocellular carcinoma (HCC) cells	[77]
	SIRT6	miR-186	Lung cancer	[78]
	SIRT7	miR-3666	NSCLC	[79]
	SIRT7	miR-3666	Breast cancer cell	[64]

**Table 5:** miRNA-mediated regulation of histone lysine methyltransferases.

Site of methyl group addition	Target gene	miRNAs	Cancer tissue or cancer cell line	Reference
H3K9	SUV39H1 (KMT1A)	miR-125a-5p	Gastric cancer	[83]
	SUV39H1 (KMT1A)	miR-125b	Hepatocellular carcinoma cells	[84]
	SUV39H1 (KMT1A)	miR-122	Hepatocellular carcinoma cells	[85]
	SUV39H2 (KMT1B)	miR-675	Liver stem cells	[86]
	G9a (KMT1C)	miR-212	Lung cancer (NSCLC)	[87]
	SETDB1 (KMT1E)	miR-381-3p	Breast cancer	[88]
H3K4 (MLL1)	Mll1 (KMT2A)	miR-22-3p	Prostatic cancer	[89]
H3K36	ASH1L (KMT2H)	miR-142	Leukemia	[90]
	ASH1L (KMT2H)	miR-142	Thyroid tumors	[91]
	KMT3B (NSD1)	miR-181a	Endometrial cancer	[92]
H4K5	SMYD3 (KMT3E)	miR-124	Cholangiocarcinoma	[93]
	SMYD3 (KMT3E)	miR-346	Hepatocellular carcinoma	[94]
H3K79	DOT1L (KMT4)	miR-133b	Colorectal cancer	[95]
H4K20	SET8 (KMT5A)	miR-502	Breast cancer	[96]
	SET8 (KMT5A)	miR-502	Ovarian cancer	[97]
	SET8 (KMT5A)	miR-502	Non-small-cell lung cancer	[98, 99]
	SET8 (KMT5A)	miR-502	Colorectal cancer	[100]
	SET8 (KMT5A)	miR-502	Non-Hodgkin's lymphoma	[101]
	SET8 (KMT5A)	miR-502	Esophageal squamous cell carcinoma	[102]
	SET8 (KMT5A)	miR-502	Clear cell renal cell carcinoma	[103]
	SET8 (KMT5A)	miR-502	Hepatocellular carcinoma	[104]
	SET8 (KMT5A)	miR-7	Breast cancer	[105]
H3K27	KMT6B (EZH1)	miR-17-5p	NSCLC	[106]
	KMT6B (EZH1)	miR-765	Breast cancer	[107]
	KMT6B (EZH1)	miR-93	Breast cancer stem cells	[108]
	KMT6B (EZH1)	miR-574-5p	Prostate cancer stem cells	[109]
	KMT6 (EZH2)	miR-101	NSCLC	[110]
	KMT6 (EZH2)	miR-101	Prostate cancer	[111]
	KMT6 (EZH2)	miR-101	renal cancer	[112]
	KMT6 (EZH2)	miR-101	Endometrial cancer	[113]
	KMT6 (EZH2)	miR-26a	Lung cancer	[114]
	KMT6 (EZH2)	miR-26a	Burkitt lymphoma	[115]
	KMT6 (EZH2)	miR-26a	Rhabdomyosarcoma	[116]
	KMT6 (EZH2)	miR-26a	Prostate cancer	[117]
	KMT6 (EZH2)	miR-26a	Nasopharyngeal carcinoma	[118]
	KMT6 (EZH2)	miR-137	Liver cancer	[119]
	KMT6 (EZH2)	miR-124	Hepatocellular cell carcinoma	[120]
	KMT6 (EZH2)	miR-138	Squamous cell carcinoma cell lines	[121]
	KMT6 (EZH2) and HDAC3	miR-31	Esophageal cancer	[125]
	KMT6 (EZH2)	miR-98	Ovarian cancer stem cells	[126]

**Table 6:** miRNA-mediated regulation of histone arginine methyltransferases.

Target gene	miRNAs	Cancer tissue or cancer cell line	Reference
PRMT1	miR-503	Hepatocellular carcinoma	[127]
PRMT4 (CARM1)	miR-195	Colorectal cancer cells	[128, 129]
PRMT4 (CARM1)	miR-424-5p	NSCLC	[130]
PRMT5	miR‐92b and miR‐96	Lymphoid cancer cell lines	[131]
PRMT5	miR-1266	Prostate cancer cell lines	[132]
PRMT9	miR-543	Osteosarcoma	[133]

**Table 7:** miRNA-mediated regulation of histone demethylases.

Demethylase groups	Target gene	miRNAs	Cancer tissue or cancer cell line	Reference
KDM1	AOF2 (LSD1)	miR-137	Endometrial cancer	[135]
	AOF2 (LSD1)	miR-302	Hepatocellular carcinoma cells	[136]
	AOF2 (LSD1)	miR-302a and miR-302b	Human head and neck squamous cell carcinomas	[137]
	AOF2 (LSD1)	miR-302	Prostate cancer	[138]
KDM2	FBXL10 (KDM2B)	miR-146b	Ovarian cancer	[139]
	FBXL10 (KDM2B)	miR-448	Gastric cancer	[140]
	FBXL10 (KDM2B)	miR-146a-5p	Cervical cancer cell lines	[141]
KDM3	JMJD1A	miR-627	Colorectal tumors	[142]
	JMJD1A	miR-155	Nasopharyngeal carcinoma	[143]
	JMJD1A	let-7c	NSCLC	[144]
	KDM3A (JMJD1A/JHDM2A)	miR-22	Ewing sarcoma	[145]
KDM4	JMJD2B (KDM4B)	miR-491-5p	Breast cancer	[146]
	JMJD2B (KDM4B)	miR-491-5p	Gastric cancer	[147]
	JMJD2C	miR-335-5p	Prostate cancer	[148]
KDM5	JARID1A (RBP2)	miR-212	Hepatocellular carcinoma	[149]
	JARID1A (RBP2)	miR-212	Gastric cancer	[150]
	JARID1B (KDM5B)	miR-363-3p	Melanoma	[151]
	JARID1B (KDM5B)	miR-137	Acute lymphoblastic leukemia cell lines	[152]
	JARID1B (KDM5B)	miR-138, miR-381-3p, and miR-486-5p	Breast cancer	[153, 154]
KDM7	PHF2	miR-221	Hepatocellular carcinoma	[155]
Arginine demethylase	JMJD6	miR-770	Non-small-cell lung cancer	[156]
	JMJD6	miR-146a and miR-193a	Melanoma	[157]

**Table 8:** miRNA-mediated regulation of histone kinases.

Kinase groups	Target gene	miRNAs	Cancer tissue or cancer cell line	Reference
AGC	RPS6KA4 (MSK2)	miR-517a	Bladder cancer cell lines	[159]
	PRKCD	miR-181a	Cervical squamous cells	[160, 161]
	PRKCD	miR-181c	Ovarian cancer tissues	[162]
	PRKCD	miR-224	Ovarian papillary serous carcinoma	[163]
	PRKCB	miR-197-3p	Gastric cancer	[164]
	RSK2	miR-634	Ovarian cancer	[165]
	PRKDC	miR-488-3p	Malignant melanoma	[166]
Nonreceptor tyrosine kinase families	JAK2	mR-543	Hepatocellular carcinoma	[167]
	JAK2	miR-216a	Pancreatic cancer	[168, 169]
	JAK2	miR-204	Breast cancer	[170]
	JAK2	miR-204	Non-small lung cancer cell lines	[171]
	JAK2	miR-375	Gastric cancer	[172, 173]
	JAK2	miR-135a	Hodgkin lymphoma	[174]
	JAK2	miR-135a	Renal cancer	[175]
	JAK2	miR-135a	Gastric carcinoma	[176]
CMGC	CDK3	miR-873	Breast cancer	[177]
	CDK3	miR-4469	Primary breast tumors	[178]
	MAP3K8	miR-589-5p	Hepatocellular carcinoma	[179]
	MAP3K8	miR-144-3p	Renal cell carcinoma	[180]
	MAP3K8	miR-509-3p	Renal cell carcinoma	[181]
	GSK3B	miR-769	Melanoma	[182]
Tyrosine kinase-like (TKL)	LIMK2	miR-135a	Bladder cancer	[183]
Other kinases family	NEK6	miR-23	Hepatocellular carcinoma	[184]
	NEK6	miR-26	Marek's disease lymphoma	[185]
	NEK6	miR-506-3p	Retinoblastoma	[186]
	AURKA	miR-124-3p	Bladder cancer	[187]
	AURKA	miR-124-3p	Glioblastoma	[188]
	AURKA	miR-32	Non-small-cell lung cancer	[189]
	AURKA	miR-137	Multiple myeloma	[190]
	AURKA	let-7	Hepatocellular cancer	[191]
	AURKA	miR-490	Hepatocellular carcinoma	[192]
	AURKA	miR-4715-3p	Gastrointestinal cancers	[193]
	BUB1	miR-490-5p	Hepatocellular carcinoma	[194]
	BUB1	miR-145-3p	Prostate cancer	[195]
CAMK	CHEK2 (Chk2)	miR-191	Osteosarcoma	[196]
	CHEK2 (Chk2)	miR-182-5p	Breast cancer	[197]
	CHEK1 (Chk1)	miR-195	Non-small-cell lung cancer	[198]
	CHEK1 (Chk1)	miR-195	Colon cancer	[199]
	CHEK1 (Chk1)	miR-497	Hepatocellular carcinoma	[200]
	CHEK1 (Chk1)	miR-145	Bladder cancer	[201]
	CHEK1 (Chk1)	miR-424	Cervical cancer	[202]
	CHEK1 (Chk1)	miR-15	Breast cancer cells	[203]
	CHEK1 (Chk1)	miR-26a	Prostate cancer	[204]
	DAPK3	miR-1307	Ovarian cancer cell lines	[205]
	AMPK	miR-451	Colorectal cancer	[206]
	AMPK	miR-25-5p	Colorectal cancer	[207]
	AMPK	miR-101	Breast cancer	[208]
	AMPK	miR-34	Prostate cancer	[209]
STE	STK4	miR-18a	Prostate cancer	[210]
	PAK2	miR-4779	Colon cancer	[211]
	PAK2	miR-216a-5p	Breast cancer	[212]
	PAK2	miR-137	Melanoma	[213]
	PAK2	miR-75p	Non-small-cell lung cancer	[214]
	PAK2	miR-134	Human ovarian cancer cells	[215]
	PAK2	miR-922	Oral squamous cell carcinoma	[216]
	PAK2	miR-26a	Hepatocellular carcinoma	[217]

**Table 9:** miRNA-mediated regulation of histone phosphatases.

Phosphatase groups	Target gene	miRNAs	Cancer tissue or cell line	Reference
PPM	PPM1D	miR-499a-5p	Osteosarcoma	[221]
PPL	PPP2CA	miR-155	Colon cancer	[222]
	PPP2CA	miR-650	Thyroid cancer	[223]
	PPP2CA	miR-130b	Glioma	[224]
	PPP2CB	miR-1246	Breast cancer	[225]
	PPP2CB	miR-129-5P	Papillary thyroid carcinoma	[226]
HAD	EYA1	miR-101	Breast cancer	[227]
	EYA1	miR-562	Sporadic Wilms' tumor	[228]
	EYA2	miR-338	Breast cancer	[229]
	EYA2	miR-30a	Breast cancer	[230]
	EYA2	miR-30a	Lung adenocarcinoma	[231]
	EYA2	miR-219a-5p	Osteosarcoma	[232]
	EYA2	miR-338-3p	Cervical cancer	[233]
	EYA3	miR-708	Ewing sarcoma	[234]
CC1	DUSP1	miR-34a	Osteosarcoma	[235]
	DUSP1	miR-202-3p	Gastric neuroendocrine neoplasm	[236]
	DUSP1	miR-324	Hepatocellular carcinoma	[237]
	DUSP1	miR-101	Hepatocellular carcinoma	[238]

**Table 10:** miRNA-mediated regulation of histone desumoylation.

Target gene	miRNAs	Cancer tissue or cell line	Reference
SENP1	miR-138	Lung cancer	[239]
SENP1	miR-133-3p	Colorectal cancer	[240]
SENP1	miR-186	Renal cell carcinoma	[241]
SENP1	miR-145-5p	Prostate cancer	[242, 243]

**Table 11:** miRNA-mediated regulation of histone ubiquitination.

Target gene	miRNAs	Cancer tissue or cancer cell line	Reference
RBX1	miR-378	Lung cancer	[244]
RBX1	miR-194	Gastric cancer	[245]
HUWE1	miR-542-5p	Osteosarcoma	[246]
RNF8	miR-214	Ovarian cancer	[247]
RNF8	miR-214	Breast cancer	[248]
RNF8	miR-622	Breast cancer	[249]
UHRF1	miR-145	Bladder cancer cell lines	[201, 250]
UHRF1	miR-124	Bladder cancer	[251]
UHRF1	miR-9	Colorectal cancer	[252]
UHRF1	miR-202	Colorectal cancer	[253]
UHRF1	miR-101	Renal cell carcinoma	[254]

**Table 12:** miRNA-mediated regulation of histone deubiquitination.

Target gene	miRNAs	Cancer tissue or cancer cell line	Reference
USP3	miR-224	Colorectal cancer	[255]
USP3	miR-224	Gastric cancer	[256]
USP7	miR-205	Hepatocellular carcinoma	[257]
USP7	miR-34a	Hepatocellular carcinoma	[258]
USP22	miR-30-5p	Non-small lung cancer cell	[259]
USP22	miR-30-5p	Colorectal cancer	[260]
USP22	miR-30-5p	Nasopharyngeal carcinoma	[261]
USP22	miR-29c	Pancreatic cancer	[262]
USP22	miR-6886-3p	Hepatocellular carcinoma	[263]
USP22	miR-4490	Gastric cancer	[264]
USP22	miR-101	Papillary thyroid carcinoma	[265]

## Data Availability

There are no raw data associated with this review article.

## Abbreviations

miR: microRNA
HDAC: Histone deacetylase
HDM: Histone demethylase
HMT: Histone methyltransferases
SNP: Single nucleotide polymorphism
NSCLC: Non-small-cell lung cancer
USP: Ubiquitin-specific protease.
